# Aging is associated with an expansion of CD49f^hi^ mammary stem cells that show a decline in function and increased transformation potential

**DOI:** 10.18632/aging.101082

**Published:** 2016-11-15

**Authors:** Qiaoxiang Dong, Hui Gao, Yuanshuo Shi, Fuchuang Zhang, Xiang Gu, Anqi Wu, Danhan Wang, Yuanhong Chen, Abhik Bandyopadhyay, I-Tien Yeh, Benjamin J. Daniel, Yidong Chen, Yi Zou, Vivienne L. Rebel, Christi A. Walter, Jianxin Lu, Changjiang Huang, Lu-Zhe Sun

**Affiliations:** ^1^ Department of Cellular & Structural Biology, University of Texas Health Science Center, San Antonio, TX 78299, USA; ^2^ School of Laboratory Medicine and Life Science, Wenzhou Medical University, University Town, Wenzhou 325035, China; ^3^ Institute of Environmental Safety and Human Health, Wenzhou Medical University, University Town, Wenzhou 325035, China; ^4^ Department of Pathology, University of Texas Health Science Center, San Antonio, TX 78299, USA; ^5^ Flow Cytometry Facility, University of Texas Health Science Center, San Antonio, TX 78299, USA; ^6^ Department of Epidemiology and Biostatistics, University of Texas Health Science Center, San Antonio, TX 78299, USA; ^7^ Cancer Therapy and Research Center, University of Texas Health Science Center, San Antonio, TX 78299, USA; ^8^ Greehey Children's Cancer Research Institute, University of Texas Health Science Center, San Antonio, TX 78299, USA

**Keywords:** mammary stem cell, neoplasia, aging, breast cancer

## Abstract

Breast cancer incidence increases during aging, yet the mechanism of age-associated mammary tumorigenesis is unclear. Mammary stem cells are believed to play an important role in breast tumorigenesis, but how their function changes with age is unknown. We compared mammary epithelial cells isolated from young and old mammary glands of different cohorts of C57BL6/J and BALB/c mice, and our findings revealed that old mammary glands were characterized by increased basal cell pool comprised of mostly CD49^fhi^ cells, altered luminal-to-basal cell ratio, and irregular ductal morphology. More interestingly, basal stem cells in old mice were increased in frequency, but showed a functional decline of differentiation and increased neoplastic transformation potential. Gene signature enrichment analysis revealed a significant enrichment of a luminal cell gene expression signature in the basal stem cell-enriched population from old mice, suggesting some luminal cells were expressing basal markers. Immunofluorescence staining confirmed the presence of luminal cells with high CD49f expression in hyperplastic lesions implicating these cells as undergoing luminal to basal phenotypic changes during aging. Whole transcriptome analysis showed elevated immune and inflammatory responses in old basal stem cells and stromal cells, which may be the underlying cause for increased CD49^fhi^ basal-like cells in aged glands.

## INTRODUCTION

Many human diseases, including cancer, increase in frequency during aging [1, 2]. Most breast cancers (80%) are diagnosed in women aged > 50 years and the incidence of invasive breast cancer increases exponentially with aging [3]. Even for women carrying BRCA1 and BRCA2 mutations with family history of familial breast cancer, aging is an important risk factor [4]. Despite this evidence pointing to aging being the number one risk factor for breast cancer, few studies have attempted to explore the underlying cellular and molecular mechanisms linking aging with clinical manifestation of breast cancer [5]. During aging, stem cells responsible for lifelong tissue maintenance and repair are susceptible to changes in their niche and genomic integrity. A recent study examining cancer risk in different tissue types suggested that stem cells are potential targets for neoplastic transformation [6]. However, little direct experimental evidence links aging stem cells to spontaneous pre-neoplastic transformation and tumorigenesis.

Murine mammary stem cells are usually identified by the mammary fat pad reconstitution assay. Earlier studies based on this transplantation assay suggest that a rare population of multipotent stem cells maintains the mammary epithelium [7, 8]. More recently, using the *in vivo* genetic labeling techniques of lineage tracing, the mammary epithelium was found to be orchestrated by both bipotent and unipotent stem/progenitor cells [9, 10]. In particular, the adult basal/myoepithelial and luminal lineages are maintained largely by their own lineage-restricted basal and luminal stem/progenitor cells under *in vivo* physiological conditions [11–13].

Mammary stem/progenitor cells have the potential to drive mammary gland development and to initiate neoplastic transformation when genetically altered [14, 15]. Gene expression profiles of different subtypes of breast cancer have been shown to correspond to the profiles of stem/progenitor-enriched epithelial cells [16, 17], which suggests that these stem/progenitor cells might be the origin of certain types of breast cancer. However, it is unknown how aging might alter these stem/progenitor cell compositions, function and transformation potential. In this study, we characterized age-related changes in the murine mammary epithelium using multiple mouse strains and provide the first direct evidence that aged mammary stem cells (MaSCs) may serve as cells of origin for aberrant mammary gland regeneration with early neoplastic lesions.

## RESULTS

### Aging was associated with an altered luminal-to-basal cell ratio, an increase in MaSC frequency and a decrease in LP frequency

**Figure 1:**
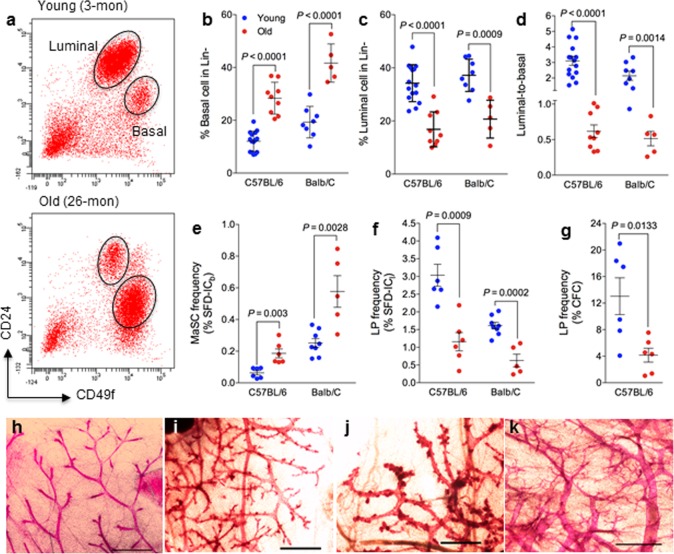
Mammary epithelial cell population and stem/progenitor cell frequency change during aging (**a**) Cells with high levels of CD49f are MaSC-enriched basal cells, and cells with high levels of CD24 are luminal progenitor-enriched luminal cells. Representative flow cytometry analysis of mammary epithelial cells from young (3 mo) and old (26 mo) virgin C57BL6/J mice. (**b-d**) Mammary epithelial cell population changes during aging. Percent basal cells (CD24^lo^CD49f^hi^) (**b**), percent luminal cells (CD24^hi^CD49f^lo^) (**c**), and luminal-to-basal cell ratio (**d**) of Lin- mammary epithelial cells isolated from young and old virgin C57BL/6 (age, 2-4 mo vs. 25-32 mo; n = 15 vs. 9) and BALB/c mice (age, 2-4 mo vs. 17 mo; n = 8 vs. 5). (**e-f**) Stem/progenitor cell frequency changes during aging. Average frequencies of MaSC (**e**) expressed as % sphere formation and differentiation-initiating cells from basal cells (SFD-IC_b_), luminal progenitor cells (**f**) expressed as % sphere formation and differentiation-initiating cells from luminal cells (SFD-IC_l_), and luminal progenitor cells (**g**) expressed as % colony forming cell (CFC) from luminal cells in young and old virgin C57BL/6 (age, 2-3 mo vs. 25-26 mo; n = 6) and BALB/c mice (age, 2-4 mo vs. 17 mo; n = 8 vs. 5). (**h-k**) Representative examples of whole mount carmine alum staining of mammary glands collected from young (**h**) and old (**i-k**) virgin C57BL6/J mice. Scale bars, 1 mm

Mammary epithelial cells include basal cells that contain MaSCs and luminal cells that contain luminal progenitor (LP) cells. To be consistent with existing literature as well as to facilitate comparison among different studies, here we used the same terminology of MaSC and LP for basal and luminal restricted stem and progenitor cells. Different marker combinations [18] have been used to identify basal and luminal cells, and fluorescence-activated cell sorting separate MaSC- and LP-enriched populations. We identified MaSC-enriched basal cells as CD24^lo^CD49f^hi^ cells that were CD31^−^CD45^−^TER119^−^ cells (also known as Lin^−^), and LP-enriched luminal cells as Lin^−^CD24^hi^CD49f^lo^ cells (Fig. 1a).

The effects of age on murine MaSCs or luminal progenitor cells were evaluated in mammary glands from young (age, 2 to 4 months) versus old C57BL/6 mice (25 to 32 months), and young (2 months) versus old BALB/c mice (17 to 27 months). Young mice at diestrus were excluded from data analysis because of the known hormonal (progesterone) effects of diestrus stage on MaSCs [19]. Similarly, old mice bearing pituitary tumors were excluded from data analysis because of the significant effect of hyperprolactinemia on mammary glands, which are usually characterized with extremely dilated primary ducts (Supplemental Fig. S1a). All old mice used in the present study were nulliparous, free of lymphoma or other tumors or wounds, and were generally in good health. We observed significantly more basal cells (Fig. 1a and b), less luminal cells (Fig. 1a and c), and subsequently a lower mean luminal-to-basal (L/B) cell ratio (Fig. 1d) in mammary glands of old mice than in those of young mice in both mouse strains. In general, old mammary glands were characterized with an L/B < 1, and young glands were characterized with an L/B > 1. We found similar changes with another set of surface markers of CD24 and CD29 using a different cohort of young (5 months) and aged C57BL6/J (27 months) (Supplemental Fig. S1b).

A sphere formation and differentiation assay [20] was used to determine whether an increase in the number of MaSC-enriched basal cells in aged mammary glands correlates with an increase in MaSC frequency. This assay consists of a suspension culture of sorted basal or luminal cells in which they form mammospheres, followed by a 3-dimensional Matrigel culture to induce sphere differentiation into solid organoids (Supplemental Fig. S1c) originated from MaSCs, and spheres that differentiated into hollow organoids (Supplemental Fig. S1d) originated from LPs. For the C57BL/6 strain, the MaSC frequency, as measured by the number of sphere formation/differentiation-initiating cells (SFD-IC), increased 3.2-fold from young (0.06% or 1 SFD-IC per 1667 epithelial cells) to old mice (0.19% or 1 SFD-IC per 526 epithelial cells), and the LP frequency decreased 2.5-fold from young (3.0% or 1 SFD-IC per 33 epithelial cells) to old mice (1.2% or 1 SFD-IC per 83 epithelial cells) (Fig. 1e-f). A similar observation was made in BALB/c mice with a 2.3-fold increase of MaSC frequency and a 2.6-fold decrease of LP frequency (Fig. 1e-f).

The LP cells can also be quantified by the number of luminal cells capable of forming discrete colonies (Supplemental Fig. S1e) when plated on an irradiated NIH3T3 cell feeder layer [21]. Using this assay, we observed an approximately 3.1-fold decrease of LP frequency from young (13%) to old C57BL/6 mice (4.2%) (Fig. 1g), which exhibited the same trend of change as the LP frequency measured using the SFD assay (Fig. 1f). However, this assay is not suitable for BALB/c mice because luminal cells from BALB/c mice gave rise to not only fewer colonies (at least 10-fold less than that of C57BL/6) but also much smaller colonies [22]. Findings from these in vitro colony formation assays demonstrated that MaSC frequency increased and LP frequency decreased with age.

It is known that the progesterone surge during the diestrus stage of the estrus cycle can dramatically increase basal cell numbers [19]. To assess whether the increased basal cells in aged glands was possibly due to effects from the estrus cycle, we first checked the estrus cycle in old mice. Following a two-week vaginal smear on a daily basis, we found that old mice (> 25 months) were not cycling and most of them rested at proestrus (Supplemental Fig. S1f). This was confirmed by a low serum progesterone level in old mice (ranging from < 0.21 ng/mL to 1.46 ng/mL, Supplemental Fig. S1g). We further examined the gland morphological appearance with whole-mount carmine staining. In comparison with young glands (Fig. 1h), we found increased tertiary branching and disorganization in glands from aged mice (Fig. 1i-k). In particular the increased tertiary branching, which resembles glands of pregnant females, may explain the reversed L/B ratio in aged glands.

### Old MaSC-regenerated mammary glands showed declining function

We evaluated self-renewal and tissue functionality of MaSCs with an in vivo serial transplantation assay. To ensure that the regenerated outgrowth was truly derived from transplanted donor cells, we used a cohort of young (7-9 months) and old (26-29 months) DsRed-C57BL/6 mice as donors. These DsRed mice exhibited similar basal and luminal epithelial cell composition as those of wild type C57BL/6 described above. Three-week old athymic female nude mice were used as recipients for in vivo transplantation because our earlier findings showed incompatibility of donor cells from DsRed mice into cleared fat pads of wild type C57BL/6 recipients. Young and old MaSCs–formed solid organoids were transplanted into the cleared fat pads of the right and left inguinal mammary glands, respectively, of the same recipient mice. Out of 15 injected cleared fat pads, we obtained similar numbers of positive outgrowths (Supplemental Fig. S2) for young (11 out of 15; 73%) and old donors (12 out of 15; 80%) (Table 1). We further isolated MaSC-enriched basal cells from the regenerated glands and used them for secondary transplants (Supplemental Fig. S2), and the engraftment was 100% for both young and old donors (Table 1). While these findings suggest no significant difference in their ability of regeneration between young and old MaSCs, further studies with limiting dilution transplantation are necessary to score their renewal capacity with more rounds of serial transplants.

**Table 1:** *In vivo* transplant of young and old MaSCs

Transplants	Donor cells	Positive / total CFP (%)	
		Young	Old
1^st^ transplants	Basal 3D organoid (1 per CFP)	11/15 (73%)	12/15 (80%)
2^nd^ transplants	Basal cells (650-1800 cells per CFP)	5/5 (100%)	5/5 (100%)

CFP: cleared fat pad; Donor cells come from DsRed C57BL6/J mice and 3-week old nude mice were used as recipients for the in vivo transplant.

**Figure 2:**
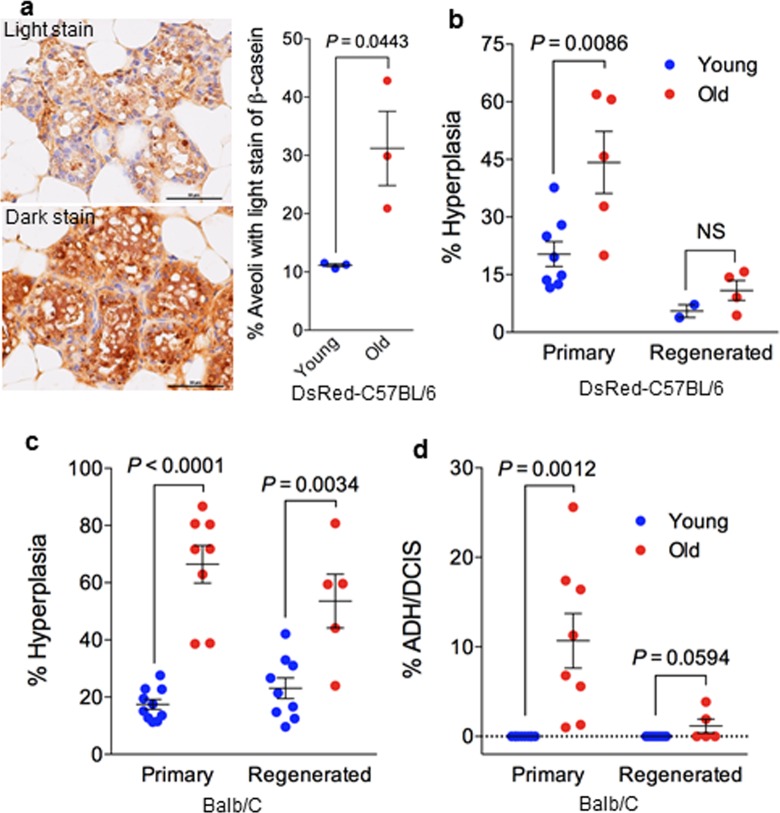
Stem cell function and transformation potential in young and old mammary glands (**a**) Immunohistochemistry staining of β-casein showing more aveoli with light staining of β-casein in regenerated outgrowths derived from old MaSCs (26-29 mon.) than in those derived from young MaSCs (7-9 mon.) isolated from DsRed-C57BL6/J donor mice (n = 3 paired regenerated outgrowths from young and old MaSCs injected in the right or left side of the same recipient mice; paired t-test). (**b**) Frequency of preneoplastic lesions (expressed as % hyperplasia) in primary and MaSC-regenerated glands from young (7-9 mon.) and old (26-29 mon.) DsRed-C57BL6/J mice. (**c**) Frequency of preneoplastic lesions (expressed as % hyperplasia) and neoplastic lesions (% atypical ductal hyperplasia/ductal carcinoma in situ [ADH/DCIS]) in primary and MaSC-regenerated glands from young (2-4 mon.) and old (17-27 mon.) BALB/c mice.

The functional capacity of luminal alveolar progenitors was next examined by their ability to differentiate and produce milk in response to ovarian hormones. We mated a subset of MaSC-transplanted recipient mice to measure the ability of the regenerated gland to produce milk (Supplemental Fig. S2). Out of 5 recipients, one did not get pregnant due to poor health although it gave rise to regenerated outgrowths in both inguinal sites, one only gave rise to regenerated outgrowth from the site injected with old MaSCs, but three recipients gave rise to outgrowths from both sites. These regenerated outgrowths along with endogenous thoracic glands from the same recipient mice were excised at postnatal day 1 (PND1), and immunohistochemistry showed that the majority of ducts had dark staining for the milk protein β-casein. The percent of lightly stained alveoli in the outgrowths regenerated from old MaSCs was significantly higher than that in the outgrowths regenerated from young MaSCs (Fig. 2a). Importantly, regenerated outgrowths from young MaSCs exhibited the same staining intensity as that of young endogenous thoracic glands (Supplemental Fig. S3a). These comparisons were made within the same recipient mice with the donor MaSCs being the only difference, thus these findings showed a decreased milk production in regenerated glands originating from old MaSCs. Further validation with whey acidic protein (WAP) revealed exact same pattern as that of β-casein (data not shown).

### Mammary glands generated by old MaSCs contained early neoplastic lesions

To investigate the role of MaSCs in mammary tumorigenesis, we performed histological examination for the presence of intraductal dysplastic lesions including atypical ductal hyperplasia (ADH) and ductal carcinoma in situ (DCIS) as shown in Supplemental Fig. S3b, which are precursors of carcinoma in rodents and humans, and can develop into palpable tumors when transplanted into hosts that have intact ovaries [23]. In this DsRed C57BL/6 cohort, we observed a higher frequency of hyperplastic lesions in the primary mammary glands from old mice (44 ± 8%, n = 5) than that from young mice (20 ± 3%, n = 8) (Fig. 2b). We also observed a similar trend of increasing hyperplastic lesions in old MaSC-regenerated glands in comparison with their young counterparts, but this was not statistically significant and likely due, in part, to the limited number of outgrowths obtained from young MaSCs in virgin recipients (Fig. 2b). The use of young recipients in the transplant assay may also help alleviate the severity of hyperplastic lesions in the regenerated glands. However, when we evaluated the same endpoint in the BALB/c cohort, we observed a significant increase in hyperplastic lesions in primary and regenerated glands from old MaSCs when compared with their young counterparts (Fig. 2c). We also observed higher frequency of ADH/DCIS in old primary glands than young primary glands in BALB/c mice, but this difference was diminished in the regenerated glands from young and old MaSCs (Fig. 2d). Since most mammospheres are formed by s single MaSC according to our earlier studies [20], these findings indicate that mammary neoplasia can originate from MaSCs and aging can increase the transformation potential of MaSCs.

### Basal cells in aged glands were enriched with luminal gene signature and contain K8 positive luminal cells

**Figure 3:**
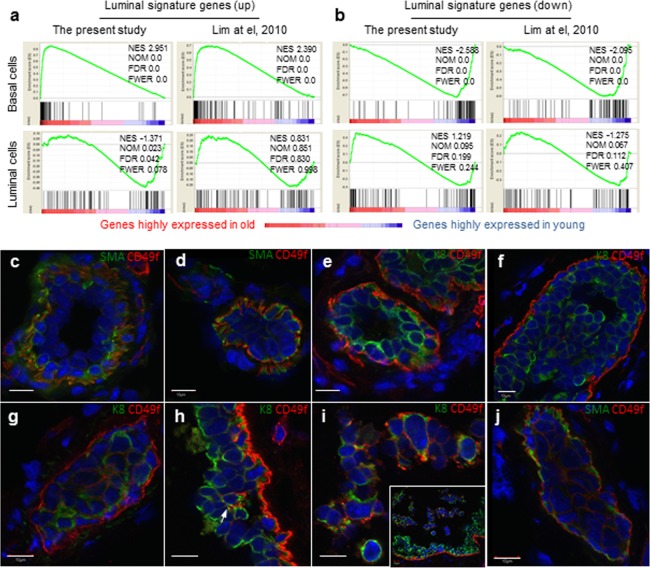
Aged basal cells were enriched with cells expressing luminal gene signature and contains K8^+^ luminal cells (**a** and **b**) Gene set enrichment analysis. Genes that are expressed in at least one sample (normalized number of reads > 1) were rank ordered according to their fold changes between young (4-6 mon.) and old (30-32 mon.), with genes highly expressed in old cells on the left. Two sets (the present study and that of Lim et al., 2010) of luminal signature genes were analyzed and indicated as black bars in the plots. The luminal cell signature genes were significantly enriched in the old basal cells, and no significant enrichment was seen in young or old luminal cells. NES, normalized enrichment score. NOM, nominal p-value. FDR, false discovery rate. FEWR, familywise error rate p-value. (**c-j**) Immunostaining of representative mammary ducts from old (26-31 mon., n = 6) C57BL6/J mice showing that basal cells were CD49f^hi^, SMA^+^ and K8^−^ in the majority normal (**c, e**) and hyperplastic ducts (**d, f**), but the presence of CD49f^hi^ luminal cells (K8^+^, SMA^−^) in a few hyperplastic lesions (**g-j**). The inset in **Panel i** shows dislodged cells from the ductal wall at a lower magnification than the main panel. Scale bars, 10 μm.

To gain insight into the age-associated molecular alterations engendering the different characteristics between old and young MaSCs, we used RNAseq to derive gene expression signatures representative of the basal, luminal, and stromal populations using freshly sorted cells and basal and luminal cell-derived spheres. To derive basal cell gene signatures, we compared the gene expression levels of young basal cells to those of young luminal cells and stromal cells. The upregulated gene signature for basal cells would be the overlapping (intersection) of genes that are higher in basal cells when compared to each of the other two types of cells (as shown in Supplemental Fig. S4). The downregulated gene signature for basal cells was derived similarly through the pairwise comparisons (Supplemental Fig. S4). We generated luminal cell gene signatures using the same method by comparing the gene expression levels of the luminal cells to those of basal and stromal of cells (Supplemental Fig. S4). These signature gene sets are included in Supplementary Table S1. We then used these signature gene sets to interrogate the expression profiles of basal and luminal cells derived from young and old mammary glands. Gene set enrichment analysis (GSEA) revealed significant enrichment of the luminal cell up-regulated signature genes in old basal cells, yet these luminal signature genes were distributed evenly across young and old luminal cells (Fig. 3a). When we used a luminal cell up-regulated signature gene set from an earlier study published by Lim and co-workers [17] to interrogate our dataset, we obtained very similar findings (Fig. 3a). In contrast, the luminal cell down-regulated signature genes from our study as well as from that of Lim and co-workers were significantly enriched in the young basal cells in comparison with the old basal cells (Fig. 3b). These findings indicated that genes normally expressed only in luminal cells were getting expressed in old basal cells. In comparison, the basal cell up-regulated signature genes were more enriched in the young basal cells and the basal cell down-regulated signature genes were more enriched in the old basal cells (Supplemental Fig. S5a). Yet, we did not observe any enrichment of the basal signature genes in young or old luminal cells as GSEA showed them to be evenly distributed across young and old basal cells (Supplemental Fig. S5a). Further, we used the luminal signature gene sets to interrogate the expression profiles of MaSC- or LP-enriched spheres. Similarly, the basal cell-derived MaSC spheres from old glands were significantly enriched with luminal up-regulated signature genes resembling the old luminal cell-derived LP spheres (Supplemental Fig. S5b), suggesting that both the primary basal cells and MaSC enriched basal spheres express luminal gene signature during aging.

The enrichment of luminal gene signature in old basal cells could be due to either the old basal cells expressing luminal markers or old luminal cells expressing high CD49f and thus being sorted into the basal cell fraction. To explore these possibilities, we co-stained basal and luminal lineage markers, smooth muscle actin (SMA) and cytokeratin 8 (K8) with CD49f. We did not observe any basal cell expressing the typical luminal marker K8, or any luminal cell expressing typical basal marker SMA either in normal or hyperplastic ducts, and CD49f^hi^ cells were mainly confined to the basal layer (Fig. 3c-f). Similar findings were obtained with another typical basal marker K5 (Supplemental Fig. S6). Further query with our RNAseq data showed that basal and luminal cells in aged glands retain typical basal (K5/K14/p63/ID4) and luminal (K8/Gata3/Elf5/Muc1) markers, respectively (data not shown). However, in some hyperplastic lesions, we observed high CD49f expression in luminal cells (Fig. 3g-i), and especially those luminal cells that form alveolar hyperplasia (Fig. 3g) or those dislodged from the ductal wall (Fig. 3i). None of these CD49f^hi^ luminal cells express the basal marker SMA (Fig. 3j), indicating that the luminal signature in old basal cells originated from luminal cells expressing high CD49f.

### Aged MaSCs and stromal cells show an elevated inflammatory response

**Figure 4:**
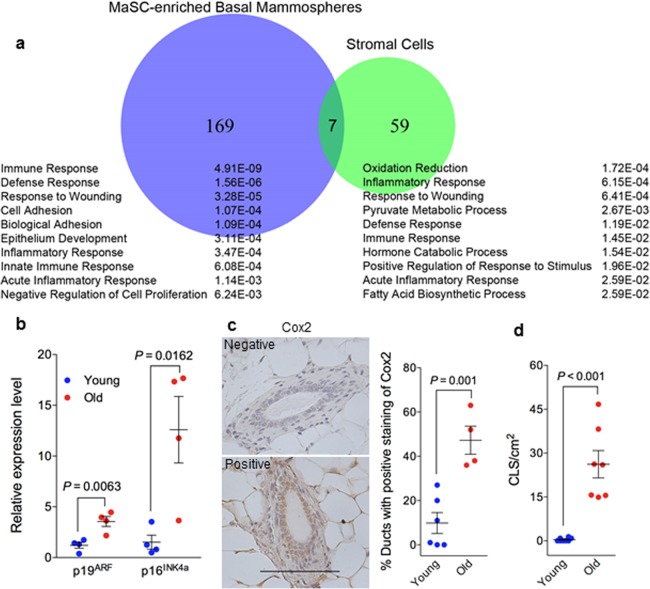
Elevated senescent phenotype and inflammatory response in old mammary glands (**a**) Venn diagram of differentially expressed genes during aging (by comparing samples from old and young mice) in MaSC-enriched basal spheres and stromal cells annotated with the top 10 enriched biological processes from Database for Annotation, Visualization and Integrated Discovery (DAVID) platform. The p value of each biological process is shown on the right. (**b**) There was greater expression of p19^ARF^ and p16^INK4a^ RNA in old stromal cells (26 mon.) than their younger (4 mon.) counterparts measured with real-time RT-PCR (n = 4). (**c**) Quantification of cyclooxygenase 2 staining in primary mammary glands from young (4 mon.; n = 6) and old (26 mon.; n = 4) C57BL/6 mice showing higher expression in old glands. The data are presented as percent ductal structures that had positive staining for cyclooxygenase 2 (defined as more than 25% cells stained positive within a particular ductal structure) in one tissue section of each mammary gland. Scale bar, 100 μm. (**d**) Quantification of macrophage crown-like structure (CLS) in primary mammary glands from young and old C57BL/6 mice showing higher numbers of CLS/cm^2^ in old (26 mon.; n = 7) than young glands (2-4 mon.; n = 8).

Whole transcriptome sequencing was performed with RNA samples from basal cell derived spheres (highly enriched in MaSCs) and stromal cells (MaSC niche), from young and old mice, to gain insights into the observed differences described above between young and old MaSCs. We identified 176 differentially expressed genes between young and old basal cell derived spheres and 66 differentially expressed genes between young and old stromal cells (Fig. 4a). Functional annotation of these differentially expressed genes with Database for Annotation, Visualization and Integrated Discovery (DAVID)[24, 25] platform showed that the primary biological processes in MaSC-enriched spheres were associated with immune, defense, and wound responses, and those in stromal cells were associated with oxidation-reduction, inflammation, and wound responses (Fig. 4a). With respect to the individual genes that are differentially expressed between young and old cells, the Cdkn2a locus, containing p19^ARF^ and p16^INK4a^, was most highly upregulated (20-fold) in the old stromal cells (adjusted *P* ≤.05), which was confirmed with real time RT-PCR assays (Fig. 4b). Cyclooxygenase 2 (Cox 2), which is not expressed under normal conditions in most cells but is elevated during inflammation, was detected at higher frequency in mammary ducts from old mice (48%) than young mice (10%) (Fig. 4c). Macrophage crown-like structures (CLS) as a hallmark of chronic inflammation was elevated in old mammary stromal (26 ± 5 CLS/cm^2^, n = 7) when compared with their young counterparts where most animals (5 out of 8) had zero incidence of CLS (Fig. 4d). Together, all these findings revealed an elevated inflammatory environment associated with old mammary glands.

## DISCUSSION

Our study showed that aging was associated with increased numbers of CD49f^hi^ basal cells, decreased numbers of luminal cells and a luminal-to-basal cell ratio of less than 1 in murine mammary epithelium. This age-related change in basal and luminal epithelial cell composition was accompanied by an increased MaSC frequency and decreased LP frequency. Morphologically, aged mammary glands displayed increased tertiary branching, irregular ductal organization, and prevalence of hyperplastic lesions. Most importantly, in vivo transplantation of MaSCs revealed significant reduction of milk production and increased hyperplastic lesions in regenerated glands derived from old, but not young BALB/c MaSCs. These findings for the first time demonstrated that spontaneous neoplastic lesions can originate from MaSCs and old MaSCs could be the susceptible targets for neoplastic transformation.

Our observations of age-associated increase in MaSC frequency and decrease in stem cell function in mammary glands from C57BL6/J and BALB/c mice are similar to findings on hematopoietic, gastrointestinal, muscle, and skin stem cells [26]. However, these results differed from findings in FVB/N mice, where no age-specific difference of mammary stem cell activity was found between old (16 to 22 months) and young mice (age, 14 weeks) [27]. We did not observe the typical aging phenotype of the altered luminal-to-basal cell ratio in mice younger than 25 months in C57BL/6. It is possible that different mouse strains may have age-related phenotypic changes at different age, as observed in C57BL6/J at age old than 25 months and BALB/c mice at age old than 17 months in the present study. In other words, different mouse strain may age at different paces, and similar age-related changes in FVB mice may occur at older ages than those that have been investigated, which was younger than 23-month-old [27]. Future studies should look into this possibility. Strain-specific differences also exist in mammary gland development [2], hormonal regulation [29], and tumorigenesis susceptibility [30]. In the present study, higher %hyperplasia was detected in aged mammary glands from BALB/c mice when compared with C57BL/6 counterparts. BALB/c mice are known to be more susceptible to radiation induced tumor formation than C57BL/6 due to their deficiency in DNA double-stranded break repair [30]. It is possible that the same mechanism is responsible for higher %hyperplasia found in BALB/c mice. Our findings of early onset of aging related MaSC changes and the prevalence of hyperplasic lesions in aged glands plus their well-known susceptibility to carcinogenesis render BALB/c mice a preferable mouse model for studying human aging and breast cancer.

Another interesting finding in the present study is the enrichment of luminal gene signature in the expanded basal cell population in old mammary glands. This phenomenon could have resulted from impure flow cytometry cell sorting from accidental inclusion of luminal cells in the basal gate. However, two measures were implemented to minimize the likelihood of this possibility. First, we always checked the cell purity post-sorting to ensure all sorted basal and luminal cells were > 99% within their designated gates. Second, with these sorted cells we performed in vitro SFD cultures and if CD24^+^CD49f^lo^ luminal cells were accidently sorted into the basal cell gate, we would see hollow organoids formed in the SFD assay (Supplemental Fig. S1d). However, all sorted basal cells gave rise to solid organoids and all sorted luminal cells gave rise to hollow organoids (Supplemental Fig. S1c-d). Therefore, the luminal signature in old basal cells is not likely due to accidental inclusion of CD24^+^CD49f^lo^ luminal cells. Rather, our immunofluorescent staining indicated possible inclusion of K8^+^CD49f^hi^ luminal cells in the basal cell fraction due to elevated CD49f in these K8 positive luminal cells, which are mainly confined to hyperplastic lesions. In normal human mammary epithelial cells, aging is associated with a reduction of myoepithelial cells and an increase in luminal cells that express K14 and CD49f, markers previously believed to be expressed exclusively in myoepithelial cells in women younger than 30 years [5]. Although it is unknown how these luminal cells obtain basal-like features during aging, they were postulated to contribute to aging-associated mammary tumorigenesis [5]. Whether these basal-like K8^+^CD49f^hi^ luminal cells in the aged murine mammary glands are the cell of origin in mammary tumorigenesis awaits further validation.

Although we observed a few luminal cells expressing high CD49f, the majority of luminal cells, either in normal or hyperplastic ducts, did not have high CD49f expression. Yet the basal cell pool in old glands was almost double the size of that in young animals. Considering the increased tertiary branching observed in old glands, we deduced that the majority of expanded CD49f^hi^ basal cells might have derived from alveolar basal cells. More recently, studies examining Timp 1-4 mutant mammary glands revealed similar observation of MaSC-enriched basal cell pool expansion during aging [31]. However, this study indicated that basal cell expansion in Timp mutants was accommodated by far greater cell density and cell compaction of mammary ducts [31]. Collectively, an expanded basal cell pool in aged mammary glands could result from increased ductal basal cells, alveolar basal cells, and K8^+^CD49f^hi^ luminal cells in hyperplastic lesions. Given the heterogeneous nature of cells in the basal population, it is difficult to discern which cell type was the main contributor to the early neoplastic lesions observed in the regenerated outgrowths derived from these old MaSCs. Although we and others have proposed possible existence of ductal and alveolar basal progenitors in the basal cell lineage [32, 33], currently there are no markers enabling the separation of these two types of basal cells. Future studies need to identify specific markers that can tag each of these distinct cell populations in the basal cell gate to allow full characterization of their roles in spontaneous tumorigenesis.

In human breast, the majority of cancer cases are estrogen receptor (ER) positive luminal subtypes [34]. Earlier studies using oncogenic transduction of isolated epithelial cells showed that basal cell derived tumors were mainly metaplastic/claudin-low ER negative [35]. Gene expression profiling also revealed that MaSC-enriched (basal) signature was concordant with claudin-low and ‘normal-like’ breast subtypes [17]. However, Blanpain's group recently reported that oncogenic *Pik3ca*^H1047R^ mutant expression at physiological levels in basal cells using keratin (K)5-CreER^T2^ mice induced the formation of luminal ER^+^ tumors, while its expression in unipotent luminal cells using K8-CReER^T2^ mice induced either liminal ER^+^ or basal-like ER^−^ tumors [36]. It is known that *PIK3CA* is the most frequently mutated gene in luminal/ER^+^ breast cancers [37–39]. Their findings suggested that the MaSC-enriched basal cells could serve as the cell of origins for luminal ER^+^ breast cancers under physiologically relevant conditions. Additionally, oncogenic *KRAS^G12D^* transduction in basal cells isolated from normal human breast tissue in a recent study produced tumors most closely resembling those of spontaneous human breast cancers classified as ‘normal-like’ or luminal A subtype, and the majority of these tumors (~88%) contained > 5% ER^+^ cells [40]. Although basal cells were found to be mostly ER negative, they were cycling during diestrus cycle when progesterone levels are high [41], revealing that basal cells can be sensitive to hormonal stimulation. Collectively, these very recent findings support our speculation that expanded basal stem cells in aged glands could serve as the cellular origins for the predominant luminal ER^+^ breast cancers observed in aged women.

Our previous study have shown that pubertal xenoestrogen bisphenol A exposure altered MaSC function, causing early neoplastic lesions in regenerated glands, which indicates that MaSCs are susceptible to xenoestrogen-induced transformation [22]. More recently, studies examining variation in cancer risk among different tissues showed strong correlations between the lifetime risk of cancers and the number of stem cell divisions [6]. These findings, together with current study, indicate that stem cells are vulnerable targets for cell transformation, and may serve as cellular origins for tumorigenesis. This may explain why the breast tissues of adolescent females are more sensitive than those of older women to the effects of ionizing radiation (IR) as this developmental stage involves extensive stem/progenitor cell proliferation and thus has a large pool of targets for IR-induced transformation [42]. Similarly, the relative susceptibility of BALB/c mice to IR or chemical induced mammary tumors comparing to C57BL/6 mice may as well attribute to the higher (3-4 fold) MaSC frequency found in BALB/c than that of C57BL/6. Correlative studies between MaSC frequency and tumor susceptibility in different developmental windows or in different mouse strains will help us further clarify the role of MaSCs in breast cancer initiation.

The expansion of basal cell population, increased MaSCs and increased tertiary branching in aged glands phenocopy mammary glands at diestrus of the estrus cycle and those at pregnancy [19, 43]. However, vaginal cytology and serum progesterone confirmed that old mice (> 25 months) had unchanging vaginal smears and low circulating progesterone levels, indicating cessation of their reproductive cycle and thus exclude possible effect from progesterone signaling. Instead, our findings revealed an elevated inflammatory response in old mammary glands, which was evidenced by activated immune/inflammatory-related gene expression and increased Cox2 and CLS. One significant source for aging associated chronic inflammation is cellular senescence [44]. Senescent cells increase with age [45, 46] and are found at sites of age-related pathologies including benign prostatic hyperplasia [47, 48]. Senescent cells also secrete pro-inflammatory cytokines, chemokines, extracellular matrix components, and growth factors, which can disrupt local tissue homeostasis and promote tumorigenesis [49]. Inoculation with premalignant or malignant epithelial cells together with senescent fibroblasts led to more and larger tumors in host mice than inoculation with the epithelial cells alone or with pre-senescent fibroblasts [50]. More recently, senescence-associated inflammatory response has been shown to contribute to colon tumorigenesis [51]. The upregulation of the *Cdkn2a* locus in old stromal cells in this study is consistent with increased p16 expression observed in many old mammalian cells including hematopoietic and neural stem cells [52, 53], suggesting a senescence phenotype in old mammary glands [49, 54]. It is worth noting that although we did not observe any difference in MaSC self-renewal between young and old, it is possible this may be due to the limited number of *in vivo* serial transplantation we did in the present study as normally 5 to 6 serial transplants are required to observe the senescence phenotype of stem cells [55]. Another significant source for aging associated chronic inflammation is an altered immune response [44]. Whole transcriptome sequencing in our study revealed an activated immune-like response in old MaSCs. Thus, aging-induced chronic inflammation might be the underlying cause for the altered epithelial cell composition, stem cell frequency and function observed in old mice. Earlier studies with epidermal and central nervous systems also support this hypothesis [56, 57].

Our findings of decreased hyperplasia incidence in regenerated glands derived from old MaSCs transplanted into cleared fat pads of young recipients when compared with primary old glands indicate the importance of stroma, where young environment may rejuvenate old stem cells [58]. Thus, elevated inflammatory environment we observed in aged stromal could play a significant role in functional changes of aged MaSCs. However, regenerated glands from aged MaSCs of BALB/c mice still exhibited > 2.3-fold increase of %hyperplasia than their young counterparts despite the use of young recipients (niche), suggesting cell intrinsic (genetic/epigenetic) impacts of aging on MaSCs. For example, recent studies in hematopoietic stem cells (HSCs) have suggested that epigenomic alterations of the DNA methylation landscape contribute to the functional decline of HSCs during aging [59]. Future studies are necessary to fully characterize both the extrinsic and intrinsic factors in MaSC aging.

In summary, our present study showed that murine MaSCs increased in frequency, decreased in function, and had increased transformation potential during normal aging. These MaSCs are susceptible to age-associated changes, and may be primed for neoplastic transformation and may subsequently serve as the cell of origin for certain subtypes of age-associated breast cancer. Future validation of this hypothesis will improve our understanding of breast cancer etiology and permit the development of novel preventive and therapeutic interventions.

## MATERIALS AND METHODS

### Animals

Animal care and use were conducted according to established guidelines approved by the Institutional Animal Care and Use Committee of the University of Texas Health Science Center, San Antonio. BALB/c mice were purchased from the National Institute of Aging repository at Charles Rivers and housed in the Nathan Shock Center clean animal facility at the Barhop Institute for Longevity Research. Wild type, CAG-DsRed. MST C57BL/6, and UBC-GFP C57BL/6 mice were originally obtained from Jackson Laboratory and raised in our facility.

### Estrus staging using vaginal smear

A vaginal flush was performed with 50 μL of sterile PBS, then spotted onto a microscope slide and allowed to air dry before immersion in 100% methanol for 1 min. Microscope slides were subsequently stained with Giemsa (1:20 v/v) for 20 min and visualized under a light microscope. Estrus staging was based on the presence and/or proportion of nucleated epithelial cells, cornified cells and lymphocytes [60]. For continuous staging in aged mice, all mice were sampled at the same time daily.

### Progesterone measurement

Blood was collected by venipuncture and allowed to clot at room temperature. Serum samples were collected by centrifugation at 2500× g for 15 min and stored at −20°C prior to analysis. Samples were shipped overnight on dry ice to the Endocrine Core at UC Davis California National Primate Research Center for progesterone analysis. Progesterone was measured by the AVIDA Centaur CP progesterone assay, a competitive immunoassay. The limit of detection was 0.21 ng/mL.

### Whole-mount Carmine staining

Mammary tissues were dissected, spread onto glass slides, and fixed overnight in Carnoy's fixative at room temperature. Following fixation, the glands were washed with 70% ethanol for 15 min, gradually rinsed in water and stained overnight in carmine alum staining solution. Stained glands were washed sequentially in 70%, 95%, and 100% ethanol twice each for 15 min. Slides were cleared in Citrisolve twice for 30 min before mounting.

### Determination of preneoplastic lesions

Preneoplastic transformation was scored by different amounts of ductal hyperplasia. Normal ductal structures were characterized by an outside myoepithelial cell layer and an inside luminal epithelial cell layer (Supplemental Fig. S3b). Hyperplastic lesions that had only a few extra layers of epithelium present were considered mild (Supplemental Fig. S3b), and those that had dilated ducts that were completely filled with uniform cells were considered severe and diagnosed as atypical ductal hyperplasia (ADH) or ductal carcinoma in situ (DCIS) when cytologic atypia and necrosis were present (Supplemental Fig. S3b).

### Immunohistochemistry

Mammary glands were fixed (24 to 48 h) in 10% neutral-buffered formalin, dehydrated in ethanol, and embedded in paraffin wax. Tissue sections (4 μm thickness) on glass slides were treated to remove the paraffin followed by a rehydration step with graded ethanol solutions. Antigen retrieval was performed by heating in sodium citrate (10 mM; pH 6.0; 95°C) for 10 min and cooling at room temperature. Endogenous peroxidase was inhibited by incubating sections with 3% H_2_O_2_ for 15 min, and nonspecific binding was blocked with 10% normal serum for 30 min at room temperature. The sections were incubated with rabbit polyclonal anti-cyclooxygenase 2 (Cell Signaling Technology, Danvers, MA) (dilution, 1:50) overnight at 4°C. Sections were washed with phosphate-buffered saline with 0.025% Triton and incubated with biotin-conjugated secondary antibodies for 1 h at room temperature. After washing, sections were incubated with streptavidin-horseradish peroxidase for 30 min and stained with diaminobenzidine for 15 min before dehydration and mounting.

### Mammary cell preparation

Mammary cells were prepared as described previously [61]. Female mice at different ages were euthanized and served as donors of mammary glands. Dissected inguinal and thoracic mammary glands (obtained from mice of indicated age) were digested in dissociation medium (1 part 10× gentle collagenase/hyaluronidase and 9 parts EpiCult-B medium [StemCell Technologies, Vancouver, Canada] supplemented with 5% fetal bovine serum) for 15 h at 37°C in a 5% carbon dioxide incubator. The organoid pellet (enriched with epithelial cells) was treated sequentially in 0.64% ammonium chloride, 0.25% trypsin and ethylenediaminetetraacetic acid, and 5 mg/mL dispase with 0.1 mg/mL DNase I. The cell suspension was filtered through a 40-micron mesh before being labeled with antibodies.

The supernatant (enriched with stromal cells) was either collected for RNAseq analysis or discarded. When collected, it was transferred to a new 50 mL centrifuge tube and centrifuge at 350× g for 5 min.

### Antibodies

Antibodies included anti-CD24 labeled with fluorescein isothiocyanate (FITC), anti-CD49f labeled with phycoerythrin (PE) or allophycocyanin (APC), anti-CD31/CD45/Ter119 mixture labeled with biotin (StemCell Technologies), and anti-CD16/CD32 (BD Biosciences, Franklin Lakes, New Jersey). APC or Pacific Blue-conjugated streptavidin (Invitrogen, Carlsbad, CA) was used to visualize the antibody mixture that was labeled with biotin.

### Cell labeling and flow cytometry

The MaSCs were enriched and isolated from endothelial (CD31) and hematopoietic (CD45 and TER119) lineage-depleted (Lin^−^) mammary epithelial cells using cell surface markers CD24 and CD49f [8]. Briefly, cells were incubated with anti-CD16/CD32 (Fcγ III/II receptor) for 10 min on ice to reduce Fc-receptor-mediated binding and then incubated on ice (15 min) with the CD31/CD45/Ter119 antibody mixture. After washing, cells were incubated with anti-CD24 labeled with FITC, anti-CD49f labeled with PE or APC (for cells from DsRed mice), and streptavidin-APC or streptavidin-Pacific Blue (for cells from DsRed mice) on ice for 10 min. After another wash, cells were sorted according to the gates illustrated in Fig. 1a using a FACSAria-IIIu (BD Biosciences).

### Sphere formation and differentiation assay

The sphere formation and differentiation (SFD) assay was performed as described previously [20]. Sorted cells were cultured in ultralow attachment 96-well plates (Corning, Midland, MI) with mouse mammosphere culture medium (EpiCult-B, StemCell Technologies) (150 μL per well) that was supplemented with 2% B27 (Invitrogen), 20 ng/mL bovine fibroblast growth factor, 20 ng/mL epidermal growth factor, 10 μg/mL heparin, 10 μg/mL insulin, 1 μg/mL hydrocortisone, and 50 μg/mL gentamicin. After suspension culture (7 days), mammospheres were counted and collected by centrifugation at 400 ×*g*. A total of 30 to 50 individual spheres were resuspended in 60 μL gel (Matrigel, BD Biosciences) for sphere differentiation. The sphere-gel drop was allowed to solidify inside a 37°C incubator for 15 min, covered with mammosphere medium supplemented with 5% fetal bovine serum, and incubated at 37°C for 9 days. The solid and hollow organoids (Supplemental Fig. S1c-d) were counted in ≥ 3 wells (approximately 120 spheres).

### 2-Dimensional colony forming cell assay

For colony formation, 1000 sorted cells were plated into each well in 6-well plates that contained mammosphere medium supplemented with 5% fetal bovine serum and irradiated NIH3T3 cells (10^4^ cells/cm^2^). After 24 h, the medium was replaced with serum-free mammosphere medium, and 8 days later the colonies were fixed with 100% cold methanol for 1 min, stained with 10% Giemsa stain for 30 min, and counted (Supplemental Fig. S1e). There were 3 replicated wells used for each sample to assess the number of colonies formed per 1000 cells.

### Stem/progenitor cell quantification

The MaSC and LP frequency was determined with the SFD assay we developed previously [20]. The SFD assay allows quantification of MaSCs by counting the number of solid organoids in 3-dimensional gel culture (Matrigel), which were shown to originate from a single MaSC. MaSC frequency was defined as percent of SFD initiating cells from the basal cell fraction out of total epithelial cells (% SFD-IC_b_). In contrast, mammospheres derived from LP-enriched luminal cells formed hollow organoids in 3-dimensional culture that were representative of LP [7, 8, 16], and LP frequency was defined as percent of SFD initiating cells from the luminal cell fraction out of total epithelial cells (% SFD-IC_l_). In addition, we also calculated LP frequency based on the frequency of colony forming cells (% colony forming cells) in 2-dimensional culture [21]. The equations were as follows:

1. Sphere formation efficiency (SFE) = No. spheres per 1,000 sorted cells
2. % SFD-IC_b_ = (SFE/1,000 × % 3-dimensional [solid]) × (% basal cells/% total epithelial cells) × 100
3. % SFD-IC_l_ = (SFE/1,000 × % 3-dimensional [hollow]) × (% luminal cells/% total epithelial cells) × 100
4. % colony forming cells = 2-dimensional colony forming cells/1,000 × (% luminal cells/% total epithelial cells) × 100

The % 3-dimensional [solid] or % 3-dimensional [hollow] in the above equations was defined as the percentage of spheres that formed a solid or hollow structure in gel culture (Matrigel). The % basal or % luminal cells was the percentage of cells gated as Lin^−^CD24^lo^D49f^hi^ (basal) or Lin^−^CD24^hi^CD49f^lo^ (luminal), and the % total epithelial cells was the sum of % cells gated as basal and luminal.

### Cleared fat pad transplant and analysis

Stem cells in the form of single solid organoids were resuspended in Hank Balanced Salt Solution (Invitrogen) with 0.2% trypan blue (Sigma, St. Louis, MO) and gel medium (50% Matrigel) (5 to 20 μL) and injected with a Hamilton syringe into the inguinal glands of 21-day-old virgin female mice cleared of endogenous epithelium. Whole mount staining was performed on fragments of the removed fat pads to ensure that the cleared fat pads were completely free of endogenous MaSCs. The presence of a rudimentary epithelial structure in the whole mount indicated the removal of the endogenous gland. FACS-sorted basal cells isolated from individually regenerated glands were used for secondary transplantation. Outgrowths, defined as epithelial structures with both ductal and lobular structures, were evaluated after 8 to 10 weeks by whole mount staining. The unique ductal growth pattern of the transplants was checked: regenerated glands from endogenous uncleared mammary ducts usually displayed unidirectional growth similar to primary glands, while regenerated glands from donor stem cells usually had bidirectional ductal growth [62]. For donor mice derived from DsRed C57BL/6, regenerated outgrowths were also checked for red fluorescent expression (Supplemental Fig. S2).

### Whole transcriptome sequencing: sample preparation and analysis

Stromal cells, sorted basal and luminal cells as well as mammospheres derived from sorted basal and luminal cells used for RNAseq analysis were two replicates pooled from a total of 8 young (4-6 months) and 6 old (26-31 months) C57BL/6 mice. Total RNA was isolated (RNeasy Mini kit, catalog number 217004, Qiagen, Venlo, Limburg, Netherlands), sequencing libraries were constructed (Illumina TruSeq RNA sample preparation protocol, catalog number RS-122-2002, Illumina, San Diego, CA), and the libraries were sequenced (HiSeq 2000, Illumina) using a single-read 50 bp sequencing protocol. The reads were aligned to the reference mouse genome (University of California Santa Cruz, build mm9) with a spliced read mapper for RNA sequencing (TopHat) [63]. Only ≤ 2 mismatches were allowed in the alignment. HTSeq-count [64] was used to count the gene expression reads and R/DESeq [65] was used to identify differentially expressed genes. Differentially expressed genes were identified with filter of Benjamini & Hochberg adjusted *P* ≤ 0.05 [66] and sequence read abundance per gene > 40% within each sample/library. Differentially expressed genes were analyzed further for functional enrichment of gene ontology by using Database for Annotation, Visualization and Integrated Discovery (DAVID) platform [24, 25].

### Gene set enrichment analysis

Gene set enrichment analysis (www.broadinstitute.org/gsea) was performed by using the 4 signatures defined above from current study as well as 4 signatures from a previously published study [17]. Default parameters were used, except that the “collapse data set” parameter was set to “true” as “GeneSymbol” and the “permutation type” parameter was set to “gene set”. Genes that are expressed in at least one sample (normalized number of reads > 1) were ranked according to their fold change between young (4-6 months) samples and old (26-31 months) samples, with genes highly expressed in old samples at the left end on the figures. Significance cutoffs were as follows: P-value ≤ 0.01 and false discovery rate ≤ 0.01.

### Statistical analysis

Differences between various treatment groups were evaluated with *t* test and Barnard exact test. Results were presented as mean ± SEM. Statistical significance was defined by *P* ≤ 0.05.

## SUPPLEMENTARY MATERIAL FIGURES AND TABLE




