# Supplementary material for: Aging is associated with an expansion of CD49fhi mammary stem cells that show a decline in function and increased transformation potential
Source: Aging (Albany NY). 2016 Nov 15;8(11):2754–70. doi: 10.18632/aging.101082 (PMC5191868; doi:10.18632/aging.101082)
Supplement: Supplementary file 1 [file aging-08-2754-s001.pdf]

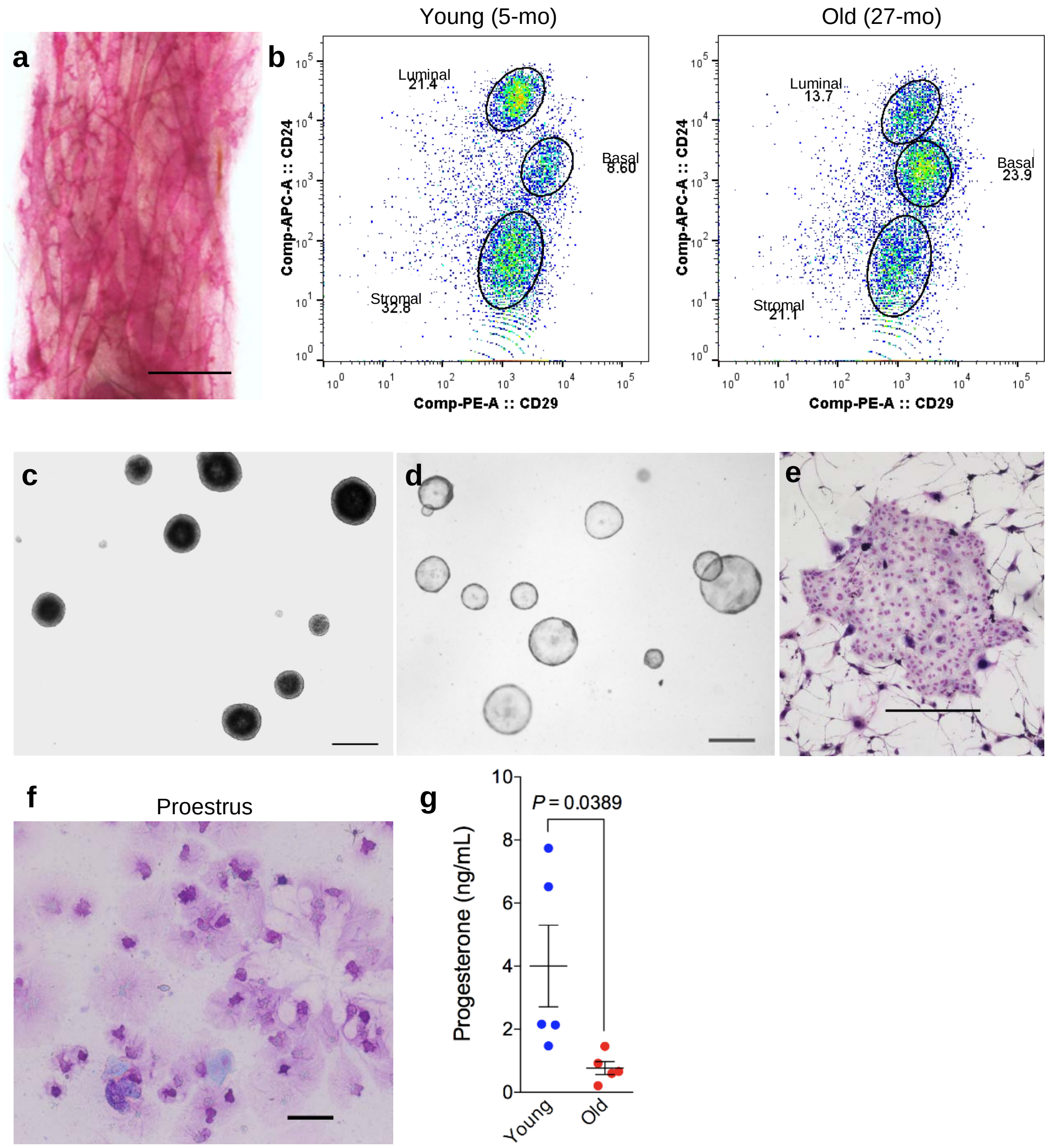

Figure S1. Whole-mount staining, flow cytometry analysis, in vitro colonies, and estrus cycle. (**a**) Whole mount carmine alum staining of mammary glands collected from old mouse bearing pituitary tumor showing dilated mammary ducts. (**b**) Flow cytometry analysis of mammary epithelial cells from young (5 mon.) and old (27 mon.) virgin GFP-C57BL6/J mice, using cell surface markers of CD24 and CD29. (**c-d**) *In vitro* colonies formed by basal MaSCs showing solid organoids (**c**) and by luminal LPs showing hollow organoids (**d**) in the sphere formation and differentiation assay. **(e)** In vitro colony formed by LP in the 2D colony forming cell assay. **(f)** Vaginal smear showing that old DsRed-C57BL6/J mice (26-29 mon.) resting at proestrus stage of the estrus cycle. **(g)** Serum progesterone level in young (9 mon.) and old (29 mon.) DsRed-C57BL6/J mice (n = 5). Scale bars, 1 mm.

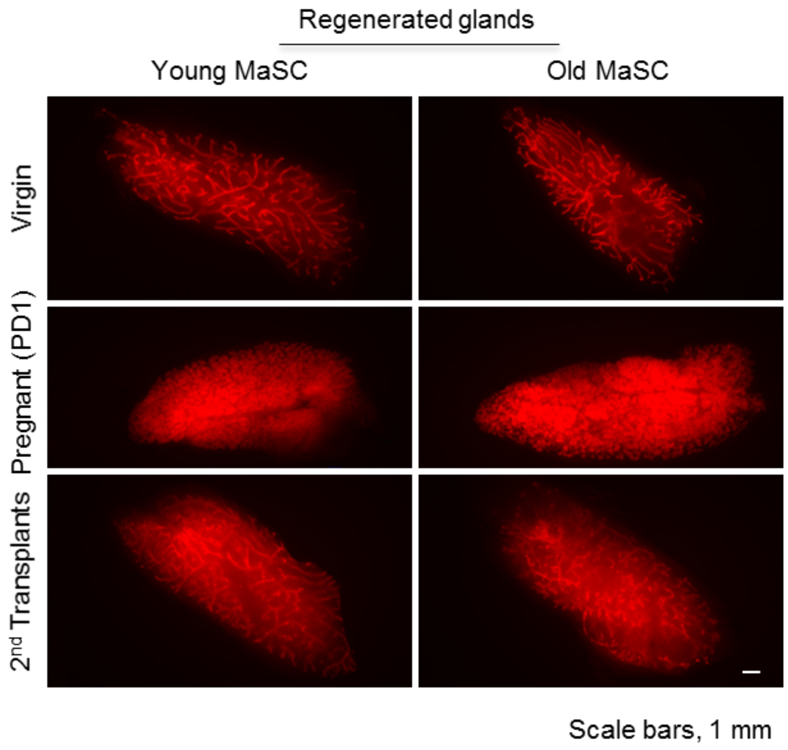

Figure S2. Whole-mount images of regenerated outgrowths formed by young and old MaSCs. Regenerated outgrowths formed by MaSCs derived from young (7-9 mon.) and old (26-29 mon.) DsRed-C57BL/6 that were injected respectively into the right and left sides of cleared fat pads of the same recipient nude mouse. Regenerated outgrowths from virgin recipients (upper panel) were terminated 10-wk after transplantation, from pregnant recipients (middle panel) were terminated at postpartum day 1 (PD1), and from virgin recipients of secondary transplant (lower panel) were terminated 10-wk after the serial transplant. Scale bars, 1 mm.

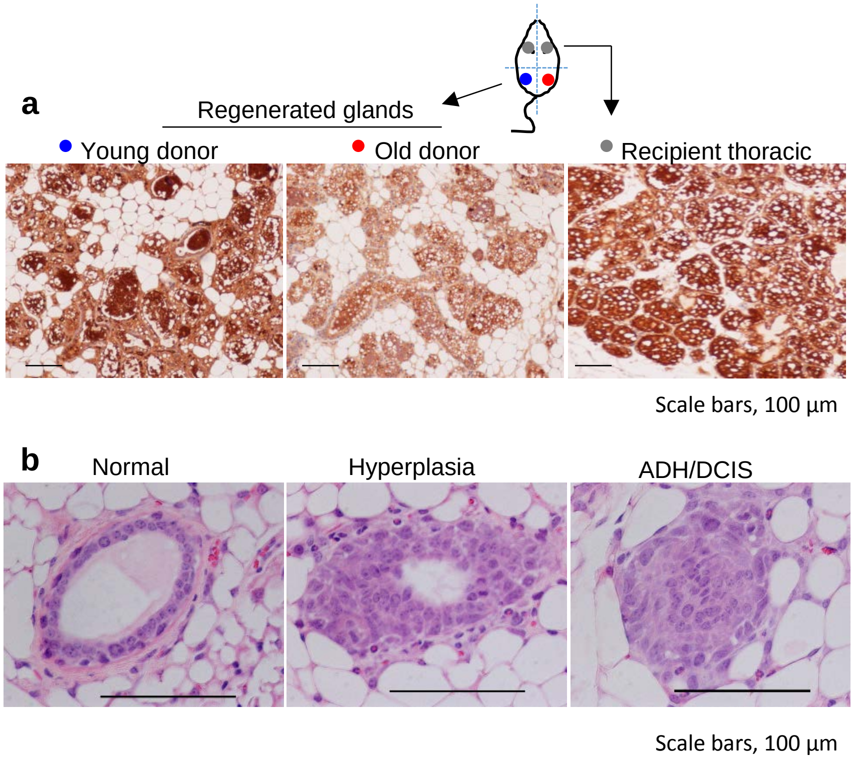

Figure S3. Immunohistochemistry analysis of milk protein and histology analysis of hyperplastic lesions. (**a**) Representative immunohistochemistry staining of β-casein in regenerated glands (postpartum day 1) formed by MaSCs isolated from young (7 mon.) and old (26 mon.) DsRed-C57BL6/J mice, along with the endogenous thoracic gland from the same recipient nude mouse. Scale bars, 100 µm. (**b**) H&E histology analysis showing normal duct, hyperplastic lesion, and atypical ductal hyperplasia/ductal carcinoma in situ (ADH/DCIS) in old (17 mon.) BALB/c mice. Scale bars, 100 µm.

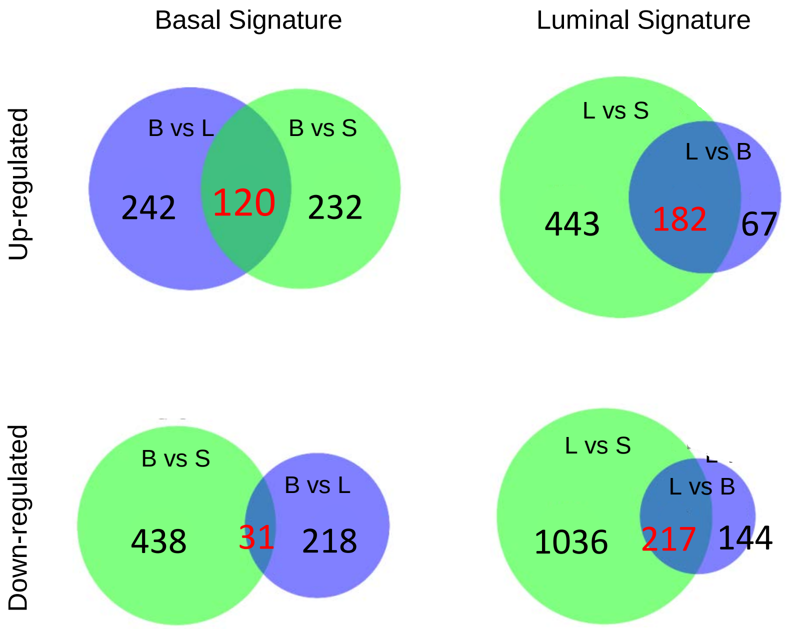

Figure S4. Pairwise comparisons in deriving gene expression signatures of basal cells and luminal cells. To derive gene expression signatures of basal cells, differential gene expression analysis was conducted in two ways: 1) comparing basal cells (B) to luminal cells (L); 2) comparing basal cells (B) to stromal cells (S). The genes that are significantly upregulated from two comparisons were overlapped and intersection were defined as basal up signature (120 genes). The intersection of significantly downregulated genes from two comparisons were defined as basal down signature (31 genes). The luminal signatures derived in the similar comparisons consist of 182 upregulated genes and 217 downregulated genes. B: basal; L: luminal; S: stromal. FDR < 0.05 was used as cutoff for filtering significantly differentially expressed genes.

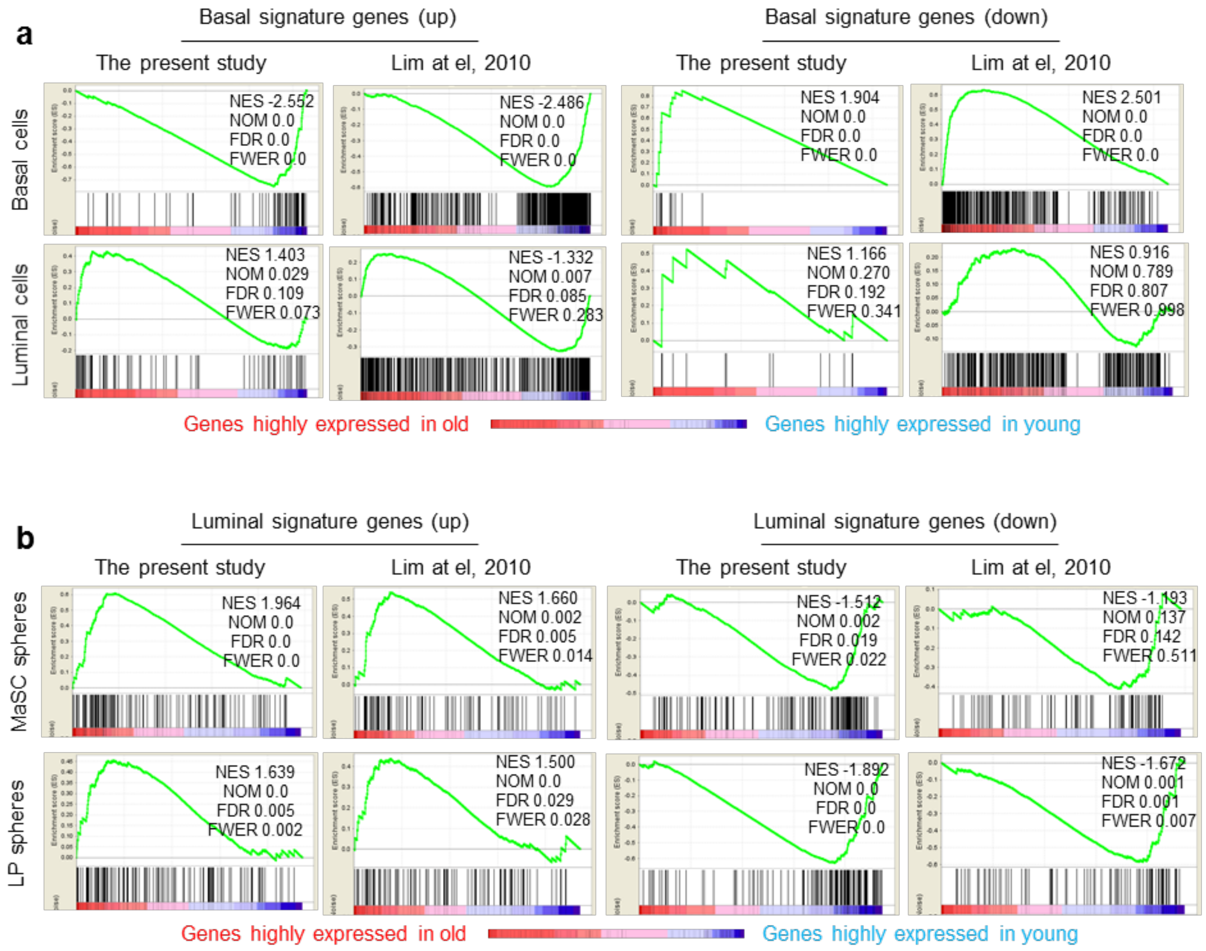

Figure S5. Gene set enrichment analysis. (**a-b**) Genes that are expressed in at least one sample (normalized number of reads > 1) were rank-ordered according to their fold changes between young (4-6 mon.) and old (30-32 mon.) samples, with genes highly expressed in old cells on the left. Two sets of basal and luminal signature genes (the present study in Supplemental Table 1 and that of Lim et al., 2010) were analyzed and indicated as black bars in the plots. The basal cell signature genes were not enriched in either young or old luminal cells (**a**), while the luminal cell signature genes were significantly enriched in the old basal spheres (**b**). NES, normalized enrichment score. NOM, nominal p-value. FDR, false discovery rate. FEWR, familywise error rate p-value.

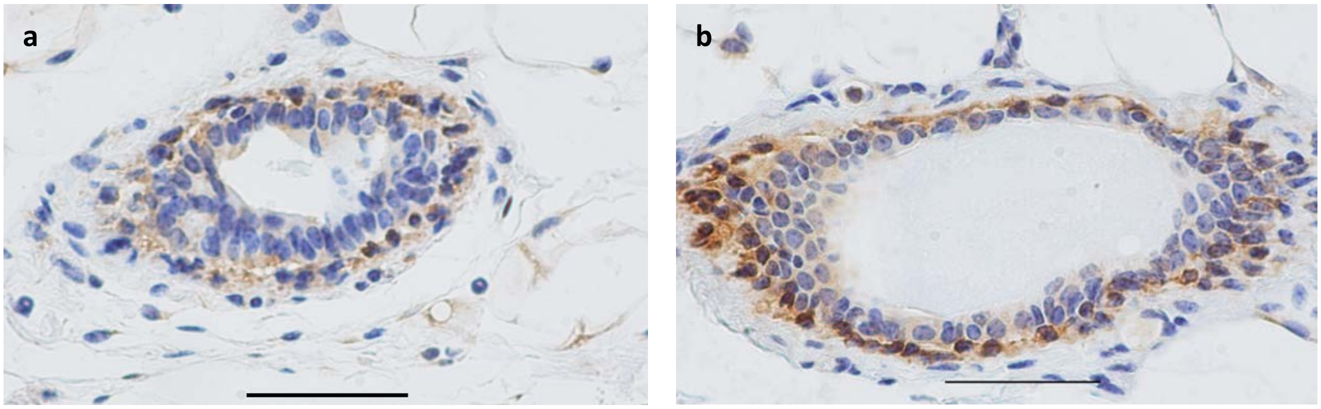

Figure S6. Immunohistochemistry staining of K5 for mammary ducts from young (a) and old(b) mice. Scale bars, 50 µm.

Supplemental Table S1. Basal and luminal signature gene sets from the present study.
Please browse the links in Full Text version of this manuscript to see Supplementary Table S1.
